# Making Sense of Carcinogens: A New Method for Navigating Mechanistic Data

**DOI:** 10.1289/ehp.124-A113

**Published:** 2016-06-01

**Authors:** Carol Potera

**Affiliations:** Carol Potera, based in Montana, also writes for *Microbe*, *Genetic Engineering News*, and the *American Journal of Nursing*.

There’s no shortage of scientific studies that link specific environmental chemicals to human cancer. Yet until recently there was no broadly accepted method to systematically organize and evaluate mechanistic data on human carcinogens, data that are key to assessing the potential carcinogenicity of unstudied chemicals. In a review published this month in *EHP*, researchers apply a new method to create order among a sprawling body of mechanistic data, based on 10 key characteristics of human carcinogens.^1^

As part of two 2012 workshops organized by the International Agency for Research on Cancer (IARC), several of the authors evaluated about 100 substances classified by IARC as Group 1 carcinogens, meaning they definitely cause human cancer.^2^ Ultimately the experts identified 10 key characteristics shared among carcinogens: the ability to 1) act as an electrophile (i.e., metabolic activation), 2) induce DNA damage and/or mutation (genotoxicity), 3) alter DNA repair or cause genomic instability, 4) induce epigenetic changes, 5) induce oxidative stress, 6) induce chronic inflammation, 7) suppress the immune system, 8) modulate receptor-mediated effects, 9) cause cell immortalization, and 10) alter a cell’s proliferation, death, or nutrient supply. Any given carcinogen will exhibit at least one of these characteristics.^1^

**Figure:**
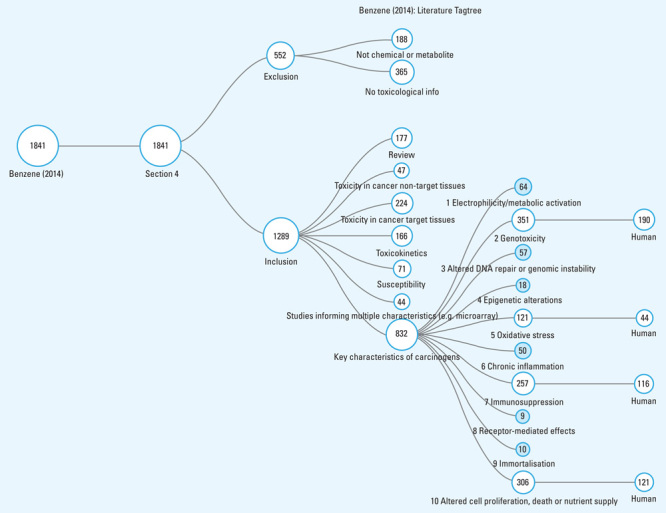
This flow chart shows how 1,841 mechanistic studies on benzene were assessed and categorized. (“Section 4” refers to the section of IARC Monographs where mechanistic data are discussed). For the sake of illustration, the authors indicated the number of human studies only for those characteristics for which more than 100 studies were identified. For 2 of the characteristics—chronic inflammation and immortalization—all of the identified studies were either null or inconclusive. The other 8 characteristics were deemed likely mechanisms for benzene. Source: Smith et al. (2016)^1^

Team members, along with colleagues at IARC and the U.S. Environmental Protection Agency, used the 10 characteristics as a framework for evaluating mechanistic data gathered for benzene and polychlorinated biphenyls (PCBs) through a systematic literature review. They focused on whether exposure to the agent induced end points associated with one or more of the key characteristics. The results were subsequently sorted by population (human, animal, or cell) or by end point.

More than 1,800 mechanistic studies exist for benzene, and almost 3,900 exist for PCBs. Applying the method yielded bodies of science supporting benzene as acting via 8 of the 10 mechanisms to cause cancer, and PCBs acting by as many as 7. It also indicated that the more volatile less-chlorinated PCBs act similarly to benzene via metabolic activation, whereas dioxin-like PCBs act primarily through receptor-mediated actions.^3^ The method has been used elsewhere to yield mechanistic portraits for the pesticides malathion (associated with 5 of the key characteristics),^4^ DDT (associated with 3 characteristics),^5^ and diazinon and glyphosate (each associated with 2 characteristics).^4^

The 10 characteristics “proved an incredibly useful tool for dividing up several thousand scientific papers into manageable amounts of information for people to review and opine on,” says first author Martyn Smith, a professor of toxicology at the University of California, Berkeley.

For many chemicals the systematic review process uncovered areas where no research had yet been conducted, which could direct future studies. For example, “information about epigenetic changes is especially lacking, but that literature is starting to build,” says Smith. Other groups are working on ways to improve the systematic literature searches using the 10 characteristics. “We need new bioinformatic tools that use machine learning to search databases more efficiently,” Smith says.

“This ground-breaking work shows us the way forward to identify and characterize key mechanistic processes across chemicals when using systematic reviews,” says Elaine Faustman, director of the Institute for Risk Analysis and Risk Communication at the University of Washington. “Until now, a clear scientific framework has been missing to allow for both identification and consistent evaluation.” Faustman was not involved in the work.

Overall, the 10 characteristics offer a consistent, objective, and systematic way to identify and evaluate human carcinogens. These insights into understanding how cancer develops will help with carcinogen hazard identification and risk assessment, and could eventually guide cancer prevention and early detection.
